# Determining Enzyme Kinetics for Systems Biology with Nuclear Magnetic Resonance Spectroscopy

**DOI:** 10.3390/metabo2040818

**Published:** 2012-11-06

**Authors:** Johann J. Eicher, Jacky L. Snoep, Johann M. Rohwer

**Affiliations:** 1 Triple-J Group for Molecular Cell Physiology, Department of Biochemistry, Stellenbosch University, Private Bag X1, Matieland, Stellenbosch 7602, South Africa; Email: eicher@sun.ac.za (J.J.E.); jls@sun.ac.za (J.L.S.); 2 Molecular Cell Physiology, Vrije Universiteit, De Boelelaan 1087, 1081 HV Amsterdam, The Netherlands; 3 Manchester Centre for Integrative Systems Biology, Manchester Institute for Biotechnology, The University of Manchester, Manchester, M60 1QD, UK

**Keywords:** NMR, enzyme kinetics, systems biology, progress curve analysis

## Abstract

Enzyme kinetics for systems biology should ideally yield information about the enzyme’s activity under *in vivo* conditions, including such reaction features as substrate cooperativity, reversibility and allostery, and be applicable to enzymatic reactions with multiple substrates. A large body of enzyme-kinetic data in the literature is based on the uni-substrate Michaelis-Menten equation, which makes unnatural assumptions about enzymatic reactions (e.g., irreversibility), and its application in systems biology models is therefore limited. To overcome this limitation, we have utilised NMR time-course data in a combined theoretical and experimental approach to parameterize the generic reversible Hill equation, which is capable of describing enzymatic reactions in terms of all the properties mentioned above and has fewer parameters than detailed mechanistic kinetic equations; these parameters are moreover defined operationally. Traditionally, enzyme kinetic data have been obtained from initial-rate studies, often using assays coupled to NAD(P)H-producing or NAD(P)H-consuming reactions. However, these assays are very labour-intensive, especially for detailed characterisation of multi-substrate reactions. We here present a cost-effective and relatively rapid method for obtaining enzyme-kinetic parameters from metabolite time-course data generated using NMR spectroscopy. The method requires fewer runs than traditional initial-rate studies and yields more information per experiment, as whole time-courses are analyzed and used for parameter fitting. Additionally, this approach allows real-time simultaneous quantification of all metabolites present in the assay system (including products and allosteric modifiers), which demonstrates the superiority of NMR over traditional spectrophotometric coupled enzyme assays. The methodology presented is applied to the elucidation of kinetic parameters for two coupled glycolytic enzymes from *Escherichia coli* (phosphoglucose isomerase and phosphofructokinase). ^31^P-NMR time-course data were collected by incubating cell extracts with substrates, products and modifiers at different initial concentrations. NMR kinetic data were subsequently processed using a custom software module written in the Python programming language, and globally fitted to appropriately modified Hill equations.

## 1. Introduction

Two polarised approaches to modeling biological systems have emerged in the literature: an inductive “top-down” approach, in which elementary interactions are inferred from general system properties; and a deductive “bottom-up” approach, which aims to predict complex systemic behaviour from a basis of mechanistically-detailed constitutive elements [1]. The “bottom-up” approach is by definition modular, allowing the integration of various sub-models into a larger systemic model (e.g., the whole-cell modelling of *Mycoplasma genitalium* [2]). This strategy has been employed in modelling various systems, including, amongst others, yeast glycolysis [3,4], sucrose accumulation in sugarcane culm [5,6], erythrocyte glycolysis [7], 2,3-bisphosphoglycerate metabolism in the erythrocyte [8], *Plasmodium falciparum* glycolysis [9], the thioredoxin system in *Escherichia coli* [10] and *Trypanosome* glycolysis [11,12].

A key requirement for the “bottom-up” approach is accurate and comprehensive kinetic data which, despite the existence of curated enzyme kinetics databases (e.g., BRENDA [13], SABIO-RK [14]), are often unavailable or inadequate for the desired experimental conditions. Experimental derivation of kinetic parameters can be expensive, labour-intensive, and often either overly simplistic and unable to comprehensively characterise enzymatic behaviour, or overly complex, having degrees of freedom that are beyond the dimensionality of experimental data [15]. Certain reaction characteristics such as reversibility and product-inhibition, and cooperative binding, which can be crucial to an *in vivo* understanding of a particular enzyme network, are at times dispensed with due to the paucity of experimental data [15]. Thus there is a need for an experimental system that is accessible and generates *in vitro* kinetic data to model the *in vivo* behaviour of enzymatic reactions comprehensively and accurately. 

An additional requirement for systems modelling is a set of simple and versatile enzyme kinetic equations. The goal of enzyme kinetic modelling has traditionally been to elucidate and represent the detailed mechanisms of enzyme-catalysed reactions, often resulting in complex kinetic equations with numerous parameters [15]. Alternatively, in an effort towards simplification, unnatural assumptions are made that often result in arbitrary parameters without a clear operational meaning [15]. The Generic Reversible Hill Equation (GRHE) overcomes these obstacles by representing cooperativity, reversibility and allosteric behaviour with a minimal set of operationally-defined parameters, making it ideal for modelling of *in vivo* biological systems [15,16]. Moreover, the kinetic parameters of the GRHE are amenable to direct experimental determination. For instance, the GRHE includes: simple half-saturation terms for substrate, product and effector binding; an *h* value representing cooperativity of binding (*h* > 1 indicates positive cooperativity, *h* < 1 negative cooperativity, and *h* = 1 absence of cooperativity); and a modifier effect parameter, α, which determines the degree of positive (α > 1) or negative (α < 1) effect of the allosteric modifier on the reaction [15,16]. 

Classical continuous enzyme assays involve collecting initial-rate data for a particular enzyme at various substrate concentrations and fitting these data to simple irreversible kinetic equations like the famous Michaelis-Menten [17,18,19] or Hill [20] equations. Less common is the alternative approach involving global fitting of complete progress curves of enzyme-catalysed reactions, instead of extracting initial rates from the first few data points and discarding the remainder of the time course [21]. This strategy involves either integration of the kinetic equation, making the substrate (and product) concentrations implicit, or differentiation of the time course data to generate rate approximations. The earliest attempts at progress-curve analysis involved the use of an integrated Michaelis-Menten equation fitted to simple single-substrate progress curves [22,23,24]. A more recent development is the closed-form solution of the integrated Michaelis-Menten equation using the Lambert-W function, which has been employed successfully for progress-curve analysis [25,26]. 

The progress-curve strategy circumvents some of the issues of traditional initial-rate enzyme kinetics, such as burst-/lag-phases altering initial velocity measurements, experimental artefacts due to coupled enzymes, and the large number of experiments that need to be performed to generate relatively little kinetic data [27]. In contrast, progress curves are acquired while the substrate and product concentrations (and possibly also the effector concentrations) are changing, therefore yielding a relatively large amount of data per experiment on the substrate and product dependence of the reaction rate as well as on interference by inhibitors [23]. However, progress curve studies can be complicated by enzyme instability, which would augment perceived product inhibition and make the analysis of time course data significantly more complex [28]. 

Initial-rate kinetic assays are classically performed by coupling the reaction of interest via interme­diate enzymes to a downstream chromogenic reaction (e.g., oxidation/reduction of NAD[P]H/NAD[P]^+^) and monitoring the increase or decrease of the chromogenic substrate using a spectrophotometer. Initial rates are approximated by fitting a tangent line to the first few data points of the time course. A highly-sensitive discontinuous variant on this protocol involves downstream coupling to a cyclical pseudo zero-order reaction and approximating substrate and product concentrations by measuring the change in cycling rate. This sensitive and relatively laborious approach has been up-scaled and mechanised using a robotic platform [29]. Alternatives to these approaches usually involve a form of labelling (e.g., radiometric labelling [30]) or chromatographic techniques (e.g., HPLC, LC-MS [31]). 

Unlike NMR spectroscopy, the approaches above are all labour-intensive, material-intensive and unable to provide direct real-time simultaneous quantification of substrate, product and effector concentrations. Moreover, recent improvements in the sensitivity of NMR spectroscopy shows that it can be an effective alternative for determination of enzyme kinetics that has been used successfully in conjunction with progress-curve analysis (e.g., assaying invertase and germacrene-D synthase [32]). ^31^P-NMR involves a suﬃciently sensitive NMR-active nucleus having an almost total natural abundance and relatively high gyromagnetic ratio, making this technique ideal for studying phosphorylated central carbon metabolites (^13^C nuclei suffer from very low natural abundance and can only be employed if costly labelled metabolites are acquired, limiting the application of ^13^C-NMR to this approach) [33]. An attractive feature of NMR spectroscopy is its applicability to *in vivo* metabolite measurements. This is a developing application, which is beginning to overcome the handicap of low sensitivity in whole organism studies (for example through the transfer of electron spin-polarisation to the nucleus of interest [34,35]). 

In this study, NMR progress curves of the two initial glycolytic reactions, phosphoglucose isomerase (PGI, EC 5.3.1.9) and phosphofructokinase (PFK, EC 2.7.1.11) in *E. coli* are acquired by incubating log-phase cell extracts with varying concentrations of substrate, product and effector. Generic Hill equations are parameterized by fitting to aggregated progress curves using a combination of genetic and least-squares algorithms. 

*E. coli* PGI catalyses the first step of glycolysis after glucose transport via the PEP:glycose phosphotransferase system, is involved in gluconeogenesis, and serves as the branch point for entry into the pentose-phosphate pathway. PGI^− ^mutants reroute flux through the pentose-phosphate pathway and exhibit markedly decreased growth rates [36]. PGI catalyses the interconversion of glucose 6-phosphate and fructose 6-phosphate and exists in two forms: the major species making up more than 90% of the activity and consisting of two subunits, the minor being a dimer of the major species [37]. PGI is derepressed under anaerobic/micro-aerobic conditions [37] and has been shown to exhibit increased activity on a shift from an aerobic to a micro-aerobic environment [38]. 

*E. coli* has two phosphofructokinases, PFK-1 and PFK-2, which catalyse the conversion of fructose 6-phosphate and ATP to fructose 1,6-bisphosphate and ADP [39]. The primary enzyme responsible for ≈ 90% of the total activity is the tetrameric PFK-1 [40]. The allosteric relationships of PFK are complex. The PFK reaction is moderately activated and inhibited under different conditions by its product ADP and other nucleoside di-and mono-phosphates, and inhibited by PEP, ATP and citrate [41,42,43,44]. At low F6P concentrations, ADP activates the reaction and reduces cooperativity; at high concentrations of F6P, ADP inhibits PFK competitively with respect to ATP (product inhibition) and non-competitively with respect to F6P [42]. PFK is strongly inhibited by phosphoenolpyruvate in a glycolytic feedback loop [42]. PFK also exhibits a degree of positive cooperative binding towards F6P, and a negative cooperativity between ATP and F6P as well as between PEP and the substrates ATP and F6P [42,44]. Many attempts at modelling the complex kinetics of PFK have been made, often employing the Monod-Wyman-Changeux model [42,45]. It is clear that arriving at a comprehensive model of PFK kinetics is no mean feat. 

**Figure 1:**
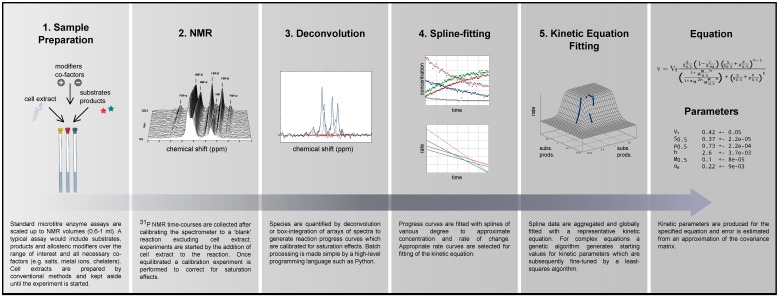
A work flow diagram.

## 2. Results and Discussion

### 2.1. Method Outline and Technical Considerations

#### 2.1.1. Method Outline

A method is presented by which kinetic equations are parameterized using NMR progress curve data for systems biological modelling (for a diagram illustrating the work flow in this study see Figure 1). Assays are prepared by combining various starting concentrations of substrate, product and effector (including all necessary co-factors and constituents for NMR spectrometry) with a cell extract containing the enzyme(s) of interest. An appropriate NMR nucleus is selected and time courses are acquired as the enzymatic reaction(s) progress towards equilibrium. Each time course is captured as an array of Free Induction Decays (FIDs), which is processed and quantified to yield a standard progress curve of concentration change over time. Progress curves are fitted independently (*i.e*., not assuming mass conservation) with splines to approximate the reaction rate by differentiating the splines at each of the measured metabolite concentrations. The spline data obtained from various runs are then globally fitted with an appropriate kinetic equation to obtain kinetic parameters. 

#### 2.1.2. ^31^P NMR Spectroscopy of Nucleoside Phosphates

**Figure 2:**
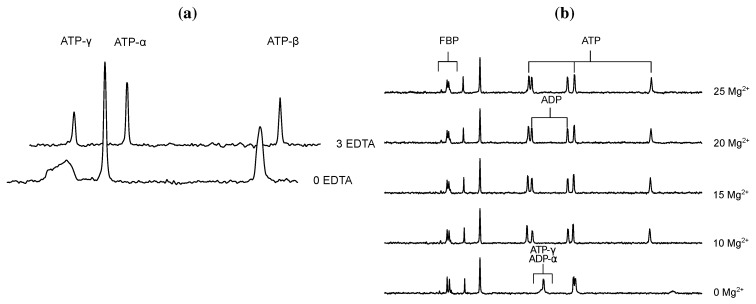
^31^P NMR (a) The effect of EDTA on the line shapes of ATP using ^31^P NMR. Spectra were collected with a 90° pulse angle and repetition time of 1 s (0.5 s acquisition time, 0.5 s relaxation delay). ATP concentration was 5 mM; (b) MgCl_2_ titration of FBP, ATP and ADP and the effect on ^31^P NMR spectral offset and line shape. Spectra were collected with a 60° pulse angle and repetition time of 1.3 s (0.8 s acquisition time, 0.5 s relaxation delay). FBP, ATP and ADP concentrations were 10 mM, and the indicated concentration of MgCl_2_ (in mM) was added. All other parameters are described in Section 3.3. Raw NMR FID data are included as supplementary material.

Using ^31^P-NMR to quantify ATP and ADP has to be approached carefully due to the diverse solvation, complexation and metal-binding behaviours of the nucleoside phosphates (see e.g., [46]). ATP binds Mg^2+^ to form the true substrate of PFK, MgATP [42]. Mg^2+^ concentration has a large effect on the NMR line shapes of ATP and ADP. Concentrations of Mg^2+^ up to and in the region of total nucleoside phosphate sharpen ATP and ADP line shapes, presumably by titrating out free nucleoside phosphate, but also shift the three resonances downfield and reduce phosphorus coupling constants [47]. However, increasing cation concentration is offset against the concomitant loss in resolution in the rest of the NMR spectrum at high concentrations (Figure 2b). Commercial preparations of ATP and ADP can contain trace amounts of metal ions, which significantly affect line shapes in an NMR spectrum and ideally should be removed by chelation before experimentation (e.g., using hydroxyquinoline or EDTA, Figure 2a). In this study, prior to the addition of cell extract, PFK assay mixtures were treated with EDTA, after which excess Mg^2+^ was added to achieve the desired experimental concentration. 

#### 2.1.3. Maximal Rate Normalisation

Due to the large number of variables involved in NMR spectrometry that are outside of human control (e.g., magnetic field inhomogeneities introduced by environmental changes such as the movement of metal items in the vicinity of the spectrometer) and the variable protein yield of cell extraction procedures (e.g., the high level of thermal denaturation introduced by sonication), it is often necessary to introduce an experimental normalisation factor to ensure that the sets of assays performed on different extracts or days remain comparable. In this study, a maximal rate assay (*i.e*., saturated with substrate(s) and in the absence of products) was performed for each experimental session or independent extract, and all associated assays were normalised to this rate. Once aggregated and globally fitted, kinetic equations were scaled by an appropriate maximal rate acquired using either NMR or an independent spectrophotometric enzyme assay (in the case of PFK in this study both NMR and coupled enzyme assays were used to determine *V*_max_). 

**Figure 3:**
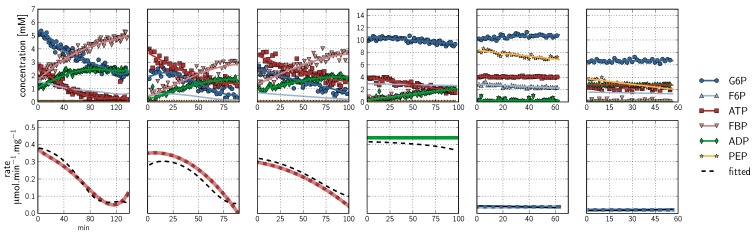
Spline fits of ^31^P-NMR Phosphofructokinase data. Data were acquired using a 90° pulse angle to collect 100 transients per FID using a repetition time of 1 s (0.5 s acquisition time, 0.5 s relaxation delay). **Top row:** Progress curves representing NMR peak integrals (G6P 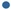, ATP 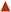, FBP 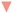, ADP 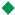, PEP 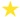) are fitted with splines (G6P 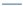, F6P 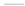, ATP 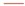, FBP 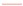, ADP 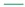, PEP 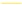). Inhibitor assays containing PEP are shown in the last two blocks. Note that with the exception of the second-last assay, F6P concentrations are inferred from equilibrium with G6P via PGI. **Bottom row**: Respective rates derived from spline-fitted NMR data. Dual colour lines indicate an average of two respective rates. For comparison, the rate calculated by the irreversible Hill equation (---, Table 1: PFK) at the specific substrate, product and effector concentrations is shown. The Hill equation parameters were the same throughout and obtained from a global fit of all the time courses shown. Rates are normalised to total protein. Raw NMR FID data, as well as NMR peak integrals and spline data, are included as supplementary material.

This approach is only valid, of course, if enzyme activities remain constant with time and there is no enzyme denaturation. As a control, we performed PFK assays at saturating substrate concentrations under conditions where these would not change significantly during the time course (Figure 3, latter three datasets). Importantly, the rates were constant over the full time course (up to 60 minutes), demonstrating a lack of enzyme denaturation. 

#### 2.1.4. Data Redundancy and Model Validation

Owing to the fact that multiple metabolites are simultaneously visible to the NMR spectrometer, NMR enzyme assays have a high data redundancy; progress curves for multiple enzymatic reactions can be generated from a single assay. In this study, progress curves used to fit the PFK reaction involved both a reverse PGI reaction and a forward PFK reaction-reactions were started with an initial concentration of F6P that was consumed in reverse by PGI and in the forward direction by PFK. PFK parameters were only fitted to data from the later stage of time courses, after PGI equilibration. This strategy provided two experimental benefits. First, lower concentrations of F6P, which are obscured by the adjacent accumulating FBP peaks, could be approximated assuming equilibrium with the easily quantifiable G6P peaks. Second, the pre-equilibration time course data could be reserved for model validation by attempting to predict the behaviour of the coupled two-enzyme system using the independently fitted parameters of both PGI and PFK in a minimal model simulation (see Section 2.3). 

**Table 1:** Kinetic Equations and Fitted Parameters for the PGI and PFK reactions.

Kinetic Equations ^a^			Fitted parameters ^b^	
PGI	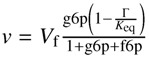 uni-uni, reversible Michaelis–Menten ^c^ [15]	*V_f_*	3.551 ± 0.050 (*V_r_* = 3.431) ^e^	μmol:min^-1^:mg^-1^
		G6P_0.5_	0.550 ± 0.236	mM
		G6P_0.5_	0.152 ± 0.017	mM
		*K*_eq_	0.286 ± 8 × 10^-6^	μmol:min^-1^:mg^-1^
PFK	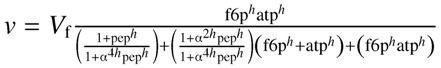 allosteric modifier: ⊝PEP bi-substrate, irreversible Hill [48]	*V_f_*	0.4435 ±0.0001	mM
		F6P_0.5_	0.4174 ±0.00006	mM
		ATP_0.5_	0.5444 ±0.0003^d^	mM
		PEP_0.5_	0.0863 ±0.0001	
		α	0.3797 ±0.0001	
		*h*	1.883 ±0.002	

*^a^* Metabolites are scaled by their half-saturation constants: e.g., 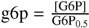 . Γ is the mass action ratio of unscaled concentrations: 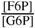; *^b^* Error is Standard Error of the Mean (S.E.M.): variance in parameter × 
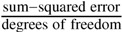
. A covariance matrix is derived from a Jacobian (a matrix of first-order partial derivatives) approximation to the Hessian matrix (a matrix of second-order partial derivatives describing the curvature of the objective function) around the solution and the associated variance in the fitted parameters is scaled by the residual variance. This correction of the estimated variance produces an unbiased estimator of the spread of the fitted parameters by scaling the residuals so that they are in units of standard deviations as described in the scipy.optimize.leastsq documentation [49]. For a fuller treatment of the Levenberg-Marquardt algorithm see Press *et al.* [50]; *^c ^*In the case of no cooperativity (*h* = 1), the reversible Hill equation reduces to the reversible Michaelis-Menten; *^d ^*Correcting for low Mg^2+ ^value as used in this study (see Results and Discussion) gives 0.1089 ± 1 × 10^−5^; *^e ^*Inferred from the Haldane relationship [15].

### 2.2. Kinetic Characterisation of Phosphoglucose Isomerase and Phosphofructokinase

#### 2.2.1. Phosphoglucose Isomerase Kinetic Parameters

##### G6P ⇌ F6P

**Figure 4:**
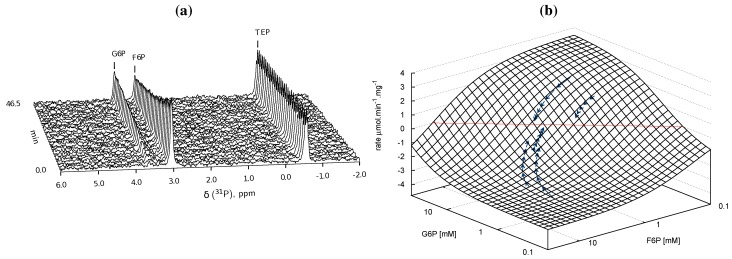
Phosphoglucose Isomerase. (a) Example of a ^31^P-NMR time course of a PGI reaction using a cell extract incubated with an initial concentration of 8.5 mM F6P and no G6P, collected at a 60° pulse angle over 47 min (0.8 s acquisition, 0.5 s relaxation). Additional NMR parameters are described in Section 3.3. In this reaction, F6P was converted in reverse to G6P as the reaction approached equilibrium. The time course is not shown to full equilibration; final concentrations were 5 and 2.8 mM for G6P and F6P respectively. TEP is an internal standard; (b) Reversible Michaelis-Menten equation (see Table 1) fitted to PGI progress curves derived from NMR data: equilibrium values are represented by the red contour line (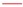), arrows indicate both the metabolite concentrations and the direction of reaction as each time course progresses towards equilibrium (→→→). The rate was normalised to total protein concentration. Substrate and product concentration axes are in logarithmic scale. R^2^= 0.99.

**Figure 5:**
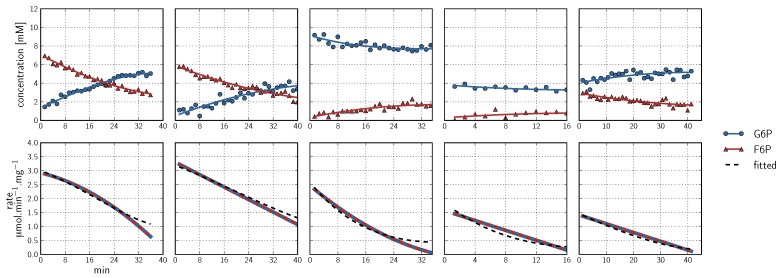
Spline fits of Phosphoglucose Isomerase data. Top row: Time courses of the PGI reaction were acquired by incubating a cell extract with various starting concentrations of substrate G6P (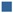) and product F6P (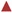) and monitoring reaction progress using ^31^P NMR with a 90° pulse angle and 1 s repetition time (1.0 s acquisition, 0.0 s relaxation) with 80 transients per FID. Other parameters are as described in Section 3.3. Progress curves derived from NMR peak integrals were fitted with splines (G6P 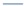, F6P 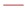). Bottom row: The respective averaged rates of the fitted splines are plotted (dual colours indicate average of two respective rates) with the rate of the fitted kinetic equation included (---, Table 1: PGI). Rates were normalised to total protein content. Raw NMR FID data, as well as NMR peak integrals and spline data, are included as supplementary material.

In order to characterise PGI, a total of five time courses in both forward and reverse directions of the reaction at different starting concentrations of substrate and product were collected, processed and fitted with splines to approximate concentrations and rates (Figure 4a and Figure 5). No “exit reactions” were observed and the reactions could be seen to proceed toward the literature equilibrium value (*K*_eq_ = 0.28 [51]). This was expected, as both enzymes adjacent to PGI, glucose 6-phosphate dehydrogenase and phosphofructokinase, require co-factors that were excluded from the assay mixture. Spline data were subsequently fitted with a uni-substrate/uni-product reversible Michaelis-Menten equation (Figure 4b, for the equation see Table 1). This equation is the reduction of the Hill equation with *h* = 1, indicating a lack of cooperativity [15]. This approach was adopted as *h* consistently fitted with a value of ≈ 1 and is additionally justified by the absence of cooperativity in literature reports regarding PGI. Fitted kinetic parameters are summarised in Table 1. 

Fitted half-saturation constants for G6P (0.550 ± 0.236 mM) and F6P (0.152 ± 0.017 mM), maximal forward rate (3.551 ± 0.050 µmol.min^−1^.mg^−1^) and the equilibrium constant (0.286 ± 8 × 10^−6^) are comparable with literature values: G6P_0.5_ 0.28 mM [52], F6P_0.5_ 0.147 mM [52], *V*f aerobic 3.29, micro-aerobic 4.66 µmol.min^−1^.mg^−1^ [38], *K*_eq_ = 0.28 [51]. The higher margin of error on G6P_0.5_ is most likely due to the sparsity of data at and below the half-saturating concentration of G6P (Figure 5). 

#### 2.2.2. Phosphofructokinase Kinetic Parameters

##### F6P + ATP ⇌ FBP + ADP

**Figure 6:**
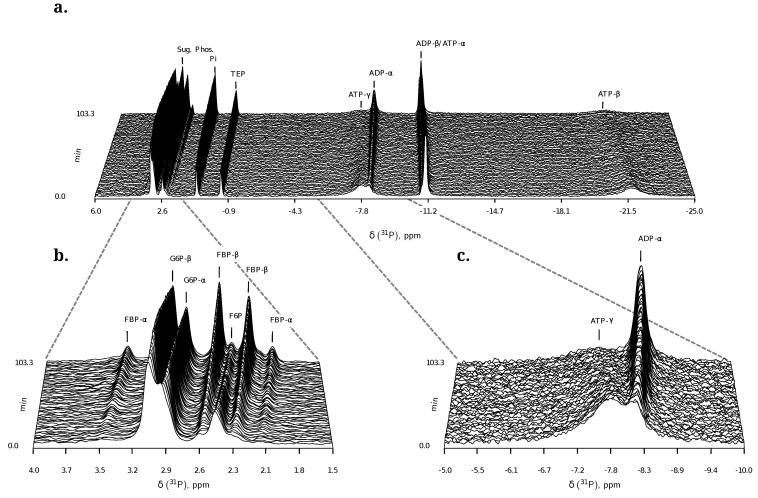
Example of a Phosphofructokinase ^31^P-NMR time course. As high Mg^2+^ concentrations can lead to line-broadening and an obscured spectrum, data were collected with no additional Mg^2+^ (beyond the trace amounts left from the growth medium) for better resolution. A pulse angle of 60° and a repetition time of 1.3 s (0.8 s acquisition time, 0.5 s relaxation delay) was used. 10 mM triethyl phosphate (TEP) is included as an internal standard. All other parameters are described in Section 3.3. (**a**) Full NMR spectrum. Initial concentrations were 14 mM G6P, 3 mM F6P, 13 mM ATP. The first few FIDs collected before the lock signal had stabilised, have been excluded; (**b**) Expansion of the sugar-phosphate region (4.0 to 1.5 ppm); (**c**) Expansion of the nucleoside phosphate region (−5 to −10 ppm).

^31^P-NMR kinetic assays for PFK were performed primarily with no proton decoupling over a relatively wide spectral width of 10 to −25 ppm to include the nucleoside phosphates (Figure 6). Six data sets were collected in total (Figure 3). As a control for lack of fructose 1,6-bisphosphatase activity, NMR was performed on a cell extract incubated with FBP at 3 and 6 mM; no activity was observed (data not shown). 

PFK NMR assays introduced a measure of complexity due to the following factors:

- Similarly to G6P, fructose 1,6-bisphosphate (FBP) exists as a pair of anomers in solution with the *β*-anomer predominating [53]. However, because of the two phosphate moieties, each anomer gives rise to two phosphorus peaks, and thus the molecule is observed as a quartet in the ^31^P-NMR spectrum (Figure 6b).
- An additional complexity is that F6P appears between the two peaks (2.6, 2.3 ppm) of the FBP *β*-anomer at ∼2.4 ppm. At low F6P and high FBP concentrations, typical of late-stage PFK time courses, F6P is obscured by the FBP peaks and has to be estimated by assuming equilibrium with the easily quantifiable G6P via the much faster PGI reaction. This is a reasonable approximation provided that the PGI reaction is allowed to equilibrate before data acquisition (the maximal rate of PGI is ∼7.5 times that of PFK). In all experiments, PGI was active, and thus to maintain higher concentrations of F6P, at times near-equilibrium concentrations of G6P were added. Data collected before PGI equilibration were excluded from fitting, reserving them for validation (Section 2.3).
- FBP-aldolase activity was not observed. This was to be expected as aldolase from *E. coli* is strictly Zn^2+^-dependent [54] and Zn^2+^ was excluded from assay mixtures.

PEP was included in two assays at concentrations of 8 and 4 mM to assay for inhibition (Figure 3). Metabolism of PEP to 2-phosphoglycerate and 3-phosphoglycerate via the enolase and phosphoglycerate mutase reactions could be observed. As the PFK reaction progressed, ADP was produced, which was subsequently consumed by pyruvate kinase, providing another exit route for PEP. The result was a significant decline in the PEP concentration over the duration of the experiment, and a concomitant maintenance of the ATP concentration (Figure 3). This dynamic concentration change is fortuitous for fitting purposes, as it can eliminate the need for performing several assays at different static effector concentrations. 

**Figure 7:**
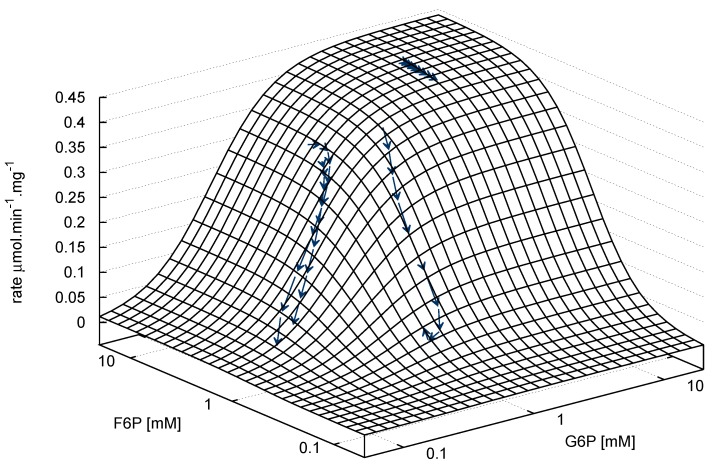
Phosphofructokinase: irreversible bi-substrate Hill equation globally-fitted to aggregated ^31^P-NMR progress curves (see Table 1 for equation and fitted parameters). Rate is normalised to total protein. Substrate concentration axes are in logarithmic scale. Arrows indicate both the metabolite concentrations and the direction of the reaction for individual time courses (→→→). PEP inhibitor assay data have been excluded from the plot as only two variables can be visualised simultaneously. R^2^= 0.96.

As a kinase and “committed step” of glycolysis, the forward reaction of PFK is favoured (*K*_eq_ = 800) [55]. In a time course assay where the reaction is allowed to equilibrate, it becomes impossible to collect data for the reverse reaction when the *K*_eq_ is too high, as such concentrations fall below the detection limits of NMR. PFK was therefore fitted with an irreversible bi-substrate Hill equation with PEP as a negative allosteric modifier (Figure 7, see Table 1 for the kinetic equation and all fitted parameters). As per standard practice for these experiments, time courses were normalised by a maximal rate for a given experimental day or cell extract, to reduce the possible introduction of error between these marginally different experimental conditions (see Section 2.1.3). The V_max_ for PFK was thus subsequently determined using a coupled enzyme assay (normalised to total protein content) and was identical to the highest NMR-determined rates. 

The maximal forward rate was determined using a spectrophotometric assay in which PFK activity was linked to NADH consumption by α-glycerophosphate dehydrogenase via fructose-1,6­bisphosphate aldolase and triosephosphate isomerase (Section 3.5). The maximal rate was determined as 0.4435 ± 0.0001 µmol.min^−1^.mg^−1^, assuming negligible activity of glyceraldehyde 3-phosphate dehydrogenase. This rate is very similar to the maximal rates determined by NMR and falls within literature range: from 0.34 (aerobic) to 0.54 (micro-aerobic) µmol.min^−1^.mg^−1^ [38,42]. 

**Figure 8:**
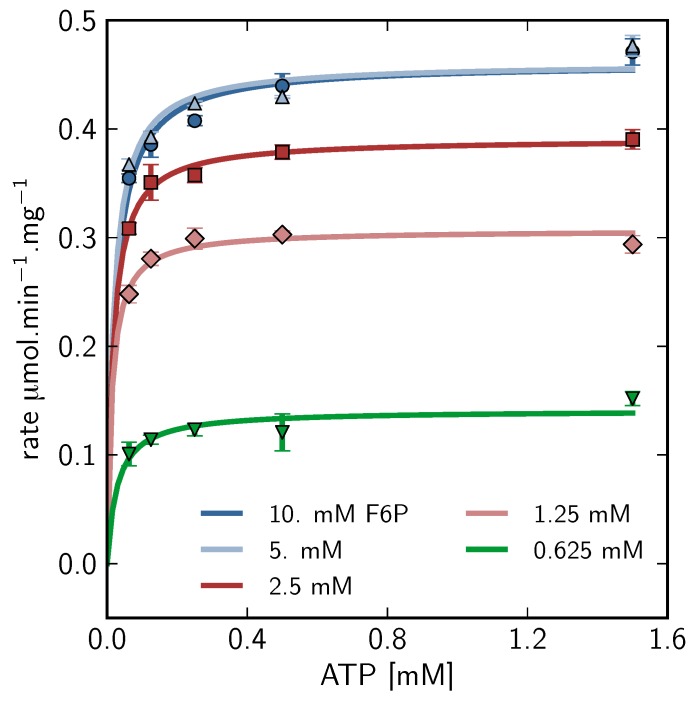
Phosphofructokinase enzyme-coupled kinetic assay. ATP saturation curves at different F6P concentrations were generated using the coupled enzyme assay system as described in Section 3.5. Points represent initial rate data and are fitted with a standard irreversible Michaelis-Menten kinetic equation. Error bars represent experimental replicates (*n* = 3) and are S.E.M. F6P: 10 mM (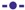), 5mM (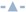), 2.5mM (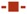), 1.25mM (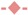), 0.625 mM (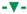).

In the absence of significant nucleoside diphosphate, the binding of F6P and ATP to PFK is that of a bi-reactant random sequential enzyme in rapid equilibrium, which displays significant antagonistic binding between the substrates [44]. Under these conditions, the binding of F6P is cooperative with a half-saturation constant of 0.35 mM at pH 8.5 [42,44]. This is very close to the fitted parameter of ≈ 0.42 mM. The fitted Hill coeﬃcient of ≈ 1.9 indicates significant cooperativity and is, as expected, lower than literature values due to the accumulation of ADP during the time course, which abolishes cooperativity; product was not allowed to accumulate in the cited studies [42,44]. ATP binding is unaffected by the presence or absence of nucleoside diphosphates. The half-saturation concentration for ATP, however, has been shown to change over the range 0.01-0.16 mM from low to high F6P concentrations, respectively [44]. The fitted ATP_0.5_ value of ≈ 0.54 mM was significantly higher than these values. For independent verification using an alternate method, a series of coupled enzyme assays was performed by varying ATP concentration over the range 0.0625-1.5 mM at five different F6P concentrations: 0.625, 1.25, 2.5, 5 and 10 mM (Figure 8). The resulting ATP half-saturation concentrations can be seen to be lower than the NMR fitted parameter values, in agreement with the literature values. 

A possible explanation for the fact that the fitted NMR parameter for ATP_0.5_ was higher than the value obtained in the coupled enzyme assay, is that the Mg^2+^ concentration was kept lower than the total ATP concentration (1 mM Mg^2+^ *vs.* 2.5-5 mM ATP) to retain resolution in the NMR spectra (see Section 2.1.2). Since the true substrate of PFK is MgATP, the effective substrate concentration was therefore significantly lower than the added ATP. Re-fitting the data assuming a five-fold lower concentration of ATP (due to saturation of available Mg^2+^ and competition for Mg^2+^ by increasing ADP concentrations) reduced the ATP_0.5_ value to 0.1089 ± 0.0001, which is well within the literature range (other parameter values showed no significant change). 

In a PFK assay system using purified enzyme with no additional reactions, one would expect products for the bi-substrate reaction (FBP and ADP) to accumulate at identical rates. However, using a whole cell extract as in the current method, unidentified background reactions could consume one or both of the products. This phenomenon was observed as a slower accumulation of ADP compared with FBP (Figure 3, first three time courses). The following proposed pathway could explain this effect:

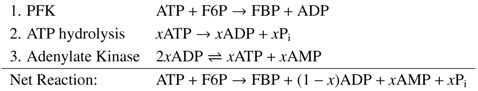


In the scheme above, background ATP hydrolysis is compensated for by the housekeeping adenylate kinase reaction, which maintains adenylates in equilibrium. Adenylate kinase will proceed in the forward direction as shown due to the initial virtually absent AMP concentration. The net reaction was observed in NMR time courses as an increase in AMP and phosphate at concentrations visibly similar to the difference between FBP and ADP. No net ADP consumption was observed in assays containing PEP, as pyruvate kinase scavenged the available ADP and phosphorylated it to produce ATP (Figure 3, latter two time courses). 

### 2.3. Method Validation: A Minimal Model of Coupled Reactions

To explore the validity of the experimental method presented here and the accuracy of the fitted kinetic parameters, the parameterized equations were evaluated by the construction of a minimal model of the 2-enzyme system under study. 

**Figure 9:**
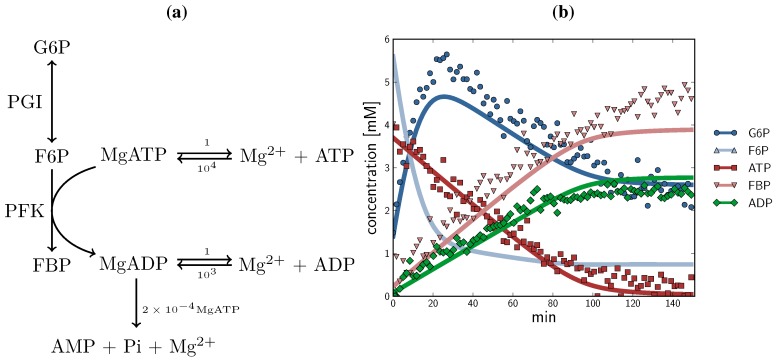
A simulation of a two-enzyme NMR time course involving PGI and PFK, beginning with initial measured concentrations. (a) Model schematic. Kinetic parameters are as described in the text. To accurately approximate experimental conditions, the model consisted of three reactions in addition to the two glycolytic enzymes: both ATP and ADP were in rapid-equilibrium reactions with MgATP and MgADP (1 mM free Mg^2+^ , *K*_eq_values were 10_4_ and 10^3 ^respectively [57,58]), and MgADP was consumed by an elementary first-order hydrolysis reaction producing AMP + Pi + Mg^2+^ (*k* = 2 × 10^−4^); see text for details; (b) Simulated time course concentrations (G6P 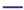, F6P 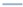, ATP 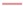, FBP 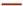, ADP 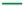) compared with experimental time course data (G6P 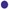, F6P 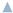., ATP 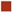, FBP 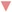, ADP 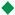). The time course started with only F6P and ATP present as substrates. Note: for parameter fitting, F6P was assumed to be in equilibrium with G6P *via* the PGI reaction, and as such no quantified F6P data are included in this figure, except for an initial concentration. AMP and orthophosphate are not shown.

A model representing an NMR time course of the PGI and PFK reactions, taking MgATP and MgADP association reactions into account, was constructed (Figure 9a) and simulated over time from measured initial concentrations (Figure 9b; time course experimental data are the same as in Figure 3, top left), using the PySCeS (Python Simulator for Cellular Systems) software [56] . The earlier portion of the time course (0-30 min, before F6P and G6P were equilibrated via the PGI reaction), which was excluded from parameter fitting, was included in this instance (see Section 2.1.4). The reaction begins with F6P and ATP; F6P is consumed in the reverse PGI reaction to produce G6P until equilibrium is reached; F6P and ATP are consumed by the forward PFK reaction. To mimic the net reaction mentioned in Section 2.2.2 and account for non-specific ATP hydrolysis, MgADP consumption was included as a first order reaction dependent on MgATP (*k* = 2 × 10^−4^, not fitted). The fitted kinetic parameters are able to predict correctly the changes in metabolite levels for this two-enzyme system, demonstrating the adequacy of the presented methodology for systems biology applications. 

### 2.4. Comparison with Other Approaches

A key methodological goal of systems biology is the development of techniques to assay enzyme kinetics *in vivo*. As a non-invasive technique, NMR technology is being developed with this application in mind and *in vivo*/*in situ* (*in situ* here refers to permeabilised cells) fluxes are becoming quantifiable [35]. Unlike traditional methods, NMR spectroscopy allows for the simultaneous observation of many different metabolites in an enzyme assay, generating a multiplicity of reaction rates determined only by the experimental starting conditions. There are several experimental benefits to this design:

(1) A single assay can be designed to produce rate and substrate concentration data for multiple enzymatic reactions, reducing time, cost and labour. In this study, a number of the datasets used in parameter fitting of the PFK reaction are time courses of both the PGI and PFK reactions.
(2) Provided an NMR-sensitive nucleus is present (^31^P in this instance), all substrates, products and effectors can be quantified in real time. This simultaneous quantification of all metabolites circumvents an important caveat of traditional enzyme kinetics. Often metabolites and effectors will be consumed or produced by ancillary reactions mediated by enzymes other than those being studied (or simply uncatalysed reactions), a phenomenon mostly invisible to traditional enzyme assay techniques. In this study, the PFK datasets exhibited this phenomenon: F6P was consumed in reverse by the preceding glycolytic enzyme PGI producing G6P; ADP, which is both a product of the PFK reaction and exhibits a complex allosteric relationship to the PFK enzyme, was consumed by a proposed hydrolytic reaction scheme, producing AMP and orthophosphate; the allosteric inhibitor PEP was consumed both in reverse by the enolase and phosphoglycerate mutase reactions producing 2-phosphoglycerate and 3-phosphoglycerate, and in the forward direction by the pyruvate kinase reaction as ADP was released from the PFK reaction, maintaining ATP levels and generating pyruvate. Though these ancillary reactions are also taking place in the NMR time course assays, they are observable and can be taken into account during the data analysis.
(3) When the concentration of an allosteric modifier changes during the experiment, this reduces the amount of data needed to fit allosteric kinetic equations by essentially providing an innate perturbation of effector concentration. In comparison, initial-rate enzyme assays require many reactions over a range of effector concentrations to achieve the same result, a diﬃculty that is exponentially compounded by the presence of multiple effectors.

Progress curve fitting using an integrated Michaelis-Menten equation (e.g., the Lambert-*W* form [25]) exhibits a number of benefits over the traditional methods (see Introduction), not least of which is the fact that the technique utilises the full time course dataset in which substrates vary in a dependent fashion, rather than merely utilising the initial rates of reactions (a notoriously diﬃcult portion of the progress curve to measure accurately [28]). However, this methodology does suffer from a number of drawbacks. Stated simply, not all of the possible causes of a change in reaction rate are distinguishable from an enzymatic time course [28]. Product inhibition is diﬃcult to account for in progress curve analyses and is circumvented only by combining the results of numerous assays or by using a technique such as NMR, which provides real-time analysis of metabolites including reaction products. This diﬃculty is compounded by the presence of multiple products as well as the lack of quantifiability of products and effectors discussed above, as the shape of the progress curve is dependent not only on gradual changes in substrate concentration, but can also be altered by changing product and effector concentrations as they are consumed or produced by invisible side reactions [23]. To assess whether a particular enzyme is suitable for progress curve analysis, a simple assay has been suggested that indicates the presence of side reactions, substrate/product inactivation and enzyme instability [59]. 

The technique of NMR spectroscopy overcomes many of these handicaps. However, it must be stressed that enzyme instability should be considered when attempting progress-curve analysis. For example, reactions should produce linear rates under saturating conditions (without significant product accumulation) over the time span of the experiment. Also, as vital kinetic information is extracted from progress curves around half-saturation concentrations and near equilibrium, NMR spectroscopy may not provide suﬃcient sensitivity to estimate all kinetic parameters associated with reactions catalysed by enzymes with extremely low half-saturation constants (< 0.5 mM) or extreme equilibrium constants. 

**Figure 10:**
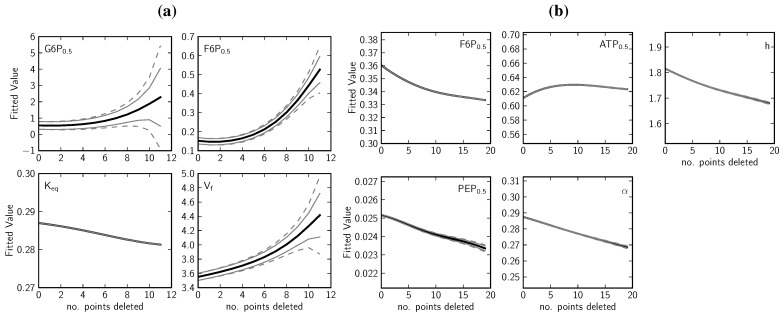
(a) Phosphoglucose Isomerase and (b) Phosphofructokinase parameter fitting performed after deleting a number of closer-to-equilibrium data points. To observe the effect of losing data from the latter part of the time courses on the fitting process, parameter fitting was performed as before using the Levenberg-Marquardt algorithm after a series of truncations had been made to the spline-fitted datasets, starting at the end of each time course, *i.e*., closer to equilibrium (original data in Figure 5 and Figure 3; fitted kinetic equations as in Table 1). Error was calculated as before (Table 1, footnote b) but with two variations: the first method involved rescaling the degrees of freedom to reflect the degree of truncation of the data (--), the second method retained the original degrees of freedom (-). This approach to error estimation was adopted to be able to distinguish between two sources of error: that due to losing data generally, and that due to specifically losing closer-to-equilibrium data. Twelve and twenty data points were deleted sequentially from the PGI and PFK data, respectively (representing up to 40 % and 24 % of the length of the longest respective time courses). Fitted parameters (-).

To investigate the effect of varying the duration of the time course during progress curve analysis, and to assess the requirement for near-equilibrium data, PGI and PFK spline-fitted data were sequentially truncated at the end of the time course and refitted with the standard fitting routine (Figure 10). The total number of truncated data points represents a portion of the closer-to-equilibrium side of each time course in the two data sets. These truncations represent up to 40% (Figure 10a) and 24% (Figure 10b) of the longest time course for PGI and PFK, respectively. Fitted parameters remained very stable after deleting at least the first half of the truncated data. It is also clear from the estimated error that as the time courses proceed towards equilibrium, the fits converge upon the parameters previously fitted and remain stable for roughly the first half of truncation. This demonstrates that the data were collected over suﬃciently long periods to include the necessary information for parameter estimation (both in terms of changing substrate and product concentrations, and in terms of the thermodynamic detail on the approach to equilibrium in the case of PGI). Truncation of the data and the subsequent explosion of the error margins on most of the PGI parameters (and the comparative stability of the PFK parameters) suggest that near-equilibrium data is indeed essential when fitting reversible kinetic equations (but of course not used for fitting irreversible equations). 

In summary, we have shown that globally fitting a collection of progress curves generated with NMR spectroscopy-a technique that allows comprehensive and simultaneous quantification of metabolites-with a generic rate equation is able to produce adequate enzyme kinetic parameters for modelling biological systems. Moreover, the method presented here overcomes most of the diﬃculties presented by traditional enzyme assays (including progress curve analyses), while being materially inexpensive, less labour-intensive and relatively rapid. 

## 3. Experimental Section

### 3.1. Growth Conditions and Media

*Escherichia coli* K12 W3110 was used to create cell stocks for kinetic assays by growing a 1 L batch culture inoculated to an optical density at 600 nm (OD_600_) of 0.1 with an overnight starter culture in M9 minimal medium [0.4% glucose, 1.28% Na_2_HPO_4_ (w/v), 0.30% KH_2_PO_4_ (w/v), 0.05% NaCl (w/v), 0.10% NH_4_Cl (w/v), 0.05% MgSO_4_ (w/v), 0.001% CaCl_2_] at 37 °C and pH 7.2, for the strict physiological control required for enzyme assays (typical constituents of rich media, such as yeast extract or tryptone digest, are highly complex and may exert any number of influences on the metabolic network). Cells were grown at pH 7.2 to buffer against the production of acidic fermentation products and to prevent premature pH-inhibition of glycolysis [60]. Cultures were gently mixed with magnetic stirrer bars during growth to produce a micro-aerobic environment. Cells were harvested in mid-log phase (OD_600_ = 0.45) and centrifuged for 10 min at 5000 rpm (∼4200 × g). Pellets were combined and resuspended in 100 mM PIPES buffer (pH 7.2) to 50 mL as a washing step and centrifuged at 5000 rpm (∼5,600 × g) for 10 min. This pellet was resuspended in 20 mL of 100 mM PIPES (pH 7.2) and separated into twenty 1 mL aliquots in Eppendorf tubes. After microcentrifugation for 10 min at 13,000 rpm (∼ 10,000 × g), the supernatant was discarded and cell pellets were frozen in liquid N_2_. These stocks were maintained at −80°C. Pellets were kept on ice between harvesting steps. 

### 3.2. Extraction

To produce a whole-cell extract, frozen cell stocks were thawed and resuspended in 1 mL 100 mM PIPES buffer (pH 7.2) with 1 mM phenylmethanesulphonylfluoride (PMSF) to inhibit serine-protease activity. Cells were extracted either by sonication or with a glass-bead shaking method (a gentler method better suited to accurate enzyme activity measurements). Protein concentration was determined by the Bradford assay method [61]. Both methods were used in this study; however, glass-bead extracts were used for velocity determination. Sonication is a faster method but suffers the drawback of thermal denaturation, which can reduce absolute maximal rate achieved by the extracted enzymes; this is unlikely to affect the kinetic parameters such as half-saturation constants. 

#### 3.2.1. Sonication

Resuspended cell pellets in a 2 mL Eppendorf tube were placed in an ice slurry and sonicated at 30 s intervals with 15 s breaks (to prevent overheating) for a total sonication time of 4 min using a micro-tip at 138 kPa. To determine the optimal sonication time that maximises protein yield without denaturing the enzymes of interest, a series of extractions was performed for various times and the activity of lactate dehydrogenase (LDH) was assayed. Optimal specific activity (* µ*mol.min^−1^.mg^−1^) was achieved at 4 min sonication time. 

#### 3.2.2. Glass-bead Extraction

Glass beads (from Sigma, 1.5 g, ≤ 106 µm diameter) were added to a 1 mL resuspended cell pellet in a 50 mL Falcon tube and rotated on an orbital shaker at 450 rpm for 1 h. This method has been shown to retain higher enzyme activity levels when compared with other methods and thus was used for maximal velocity measurements [62]. Protein and activity yield was optimised using the LDH assay. 

### 3.3. NMR Spectroscopy

All reaction components were from Sigma-Aldrich (except ATP from Boehringer-Mannheim) and prepared in 100 mM PIPES (pH 7.2, corrected by the addition of 10 M NaOH). Triethyl phosphate (TEP) was introduced as an internal standard due to its metabolic inertness [63]. A standard assay in a 5 mm glass tube was composed of: 50 mM TEP, 100 µL D_2_O, 100 µL cell extract, 100 µL per substrate/product/co-factor, 1 mM Mg^2+^ , and filled to a final volume of 1 mL with 100 mM PIPES (pH 7.2) in a 5-mm NMR tube. Initially, cell extract was excluded. This blank was used to tune the spectrometer, acquire a lock signal and shim the instrument before the reaction was started by removing the tube from the instrument, adding 100 µL cell extract, mixing several times by inverting, and re-inserting the tube. Data acquisition was initiated once a stable lock signal was achieved. 

^31^P NMR was performed at 25 °C and a frequency of 242.87 MHz on a Varian 600 MHz spectrometer with a 1 s repetition time (1.0 s acquisition/0.0 s relaxation for PGI, 0.5 s acquisition/0.5 s relaxation for PFK) to collect 80 (PGI) or 100 (PFK) transients per FID using a pulse angle of 90° with either no proton decoupling or a low power decoupling (Waltz-16) to prevent overheating of the sample. In rapid-sampling NMR, it is typically not possible to accommodate the full spin-lattice relaxation of the nucleus of interest. T1 relaxation times were determined for the metabolites in this study and varied from 0.2 s (nucleoside phosphates) to 6 s (TEP). Species concentrations were thus calibrated for incomplete relaxation with a fully relaxed spectrum (30 s relaxation time, 5 × the longest T1) of a cocktail of metabolites of interest. 

Time courses collected using a particular cell extract or on a certain day were normalised by a representative maximal rate at saturating substrate concentrations from that NMR session. This was done in order to prevent the introduction of error between days and extracts. 

Where ATP and ADP were unquantifiable due to metal ion contamination, 1.5 mM EDTA and 2.5 mM MgCl_2_ were added sequentially to a final effective concentration of 1 mM Mg^2+^ . 

### 3.4. Data Processing

All data processing was performed using a custom software module (software available from authors on request) written in the Python programming language (the Varian data import function was taken from [64]; released under the Open Software Initiative and the New BSD Licence). Spectra were processed with an exponential line-broadening function of 8.5 Hz and peaks were quantified either by box-integration or deconvolution through fitting with Lorentzian functions. Splines were fitted to concentration data using the scipy.interpolate module [49]. All fitting procedures were performed using either a custom genetic algorithm or the Levenberg-Marquardt least-squares algorithm as employed in the scipy.optimise.leastsq module [49]. Images were produced using Matplotlib [65], Gnuplot [66] and Inkscape [67]. 

### 3.5. Enzyme Assays

A coupled enzyme assay system was used for PFK with the following composition: 0.625-10 mM F6P, 0.062-1.5 mM ATP, 0.2 mM NADH, 10 mM MgCl_2_ , 1.5 U mL^−1^ FBP-aldolase, 5 U mL^−1^ triose-phosphate isomerase, 4.3 U mL^−1^α-glycerophosphate dehydrogenase. NADH consumption by α-glycerophosphate dehydrogenase was monitored at a wavelength of 340 nm in a 96-well plate reader (VarioSkan Microplate Reader, Thermo Electron Corp.; Greiner Bio-one Flat-bottom microplate) with the temperature maintained at 25°C. Activities were normalised with protein content determined by the Bradford assay [61]. All assays were performed in triplicate in 100 mM PIPES buffer at pH 7.2. 

## Supplementary Materials

**pgi fids.zip** ZIP-file containing the raw Varian NMR FID data for all the PGI runs, plus a text listing of the various experiments.

**pfk fids.zip** ZIP-file containing the raw Varian NMR FID data for all the PFK runs, plus a text listing of the various experiments.

**supp data.ods** ODS-spreadsheet containing the integrated NMR peak data for all the experiments, as well as the fitted spline data for the concentrations and rates.

## Supplementary Files

Supplementary File 1 ZIP-Document (ZIP, 4784 KB)
